# NY-ESO-1 antigen expression and immune response are associated with poor prognosis in MAGE-A4-vaccinated patients with esophageal or head/neck squamous cell carcinoma

**DOI:** 10.18632/oncotarget.26323

**Published:** 2018-11-13

**Authors:** Shugo Ueda, Yoshihiro Miyahara, Yasuhiro Nagata, Eiichi Sato, Taizo Shiraishi, Naozumi Harada, Hiroaki Ikeda, Hiroshi Shiku, Shinichi Kageyama

**Affiliations:** ^1^ Department of Gastroenterological Surgery and Oncology, Kitano Hospital, Kita-ku, Osaka 530-8480, Japan; ^2^ Department of Personalized Cancer Immunotherapy, Mie University Graduate School of Medicine, Tsu, Mie 514-8507, Japan; ^3^ Center for Comprehensive Community Care Education, Nagasaki University Graduate School of Biomedical Sciences, Nagasaki 852-8523, Japan; ^4^ Department of Pathology, Institute of Medical Science, Tokyo Medical University, Shinjuku-ku, Tokyo 160-0023, Japan; ^5^ Department of Oncologic Pathology, Mie University Graduate School of Medicine, Tsu, Mie 514-8507, Japan; ^6^ United Immunity, Co., Ltd., Tsu, Mie 514-8507, Japan; ^7^ Department of Oncology, Nagasaki University Graduate School of Biomedical Sciences, Nagasaki 852-8523, Japan; ^8^ Departments of Immuno-Gene Therapy and Personalized Cancer Immunotherapy, Mie University Graduate School of Medicine, Tsu, Mie 514-8507, Japan; ^9^ Department of Immuno-Gene Therapy, Mie University Graduate School of Medicine, Tsu, Mie 514-8507, Japan

**Keywords:** cancer vaccine, MAGE-A4, NY-ESO-1, immune response, esophageal cancer

## Abstract

MAGE-A4 antigen is a cancer-testis antigen that is frequently expressed in tumor tissues. Cholesteryl pullulan (CHP) is a novel antigen delivery system for cancer vaccines. This study evaluated the safety, immune responses and clinical outcomes of patients who received a CHP-MAGE-A4 vaccine. Twenty-two patients with advanced or metastatic cancer were enrolled, and were subcutaneously vaccinated with either 100 μg or 300 μg of CHP-MAGE-A4. Seven and 15 patients, respectively, were repeatedly vaccinated with 100 μg or 300 μg of CHP-MAGE-A4; patients in both groups received a median of 7 doses. No serious adverse events related to the vaccine were observed. Of 7 patients receiving the 100 μg dose, 2 (29%) showed immune responses, compared with 3 of the 14 (21%) patients who received the 300 μg dose. In total, MAGE-A4-specific antibody responses were induced in 5 of 21 (24%) patients. No differences in survival were seen between patients receiving the 100 μg and 300 μg doses, or between immune responders and non-responders. Eleven (50%) patients had pre-existing antibodies to NY-ESO-1. In 16 patients with esophageal or head/neck squamous cell carcinoma, the survival time was significantly shorter in those who had NY-ESO-1-co-expressing tumors. Patients with high pre-existing antibody responses to NY-ESO-1 displayed worse prognosis than those with no pre-existing response. Therefore, in planning clinical trials of MAGE-A4 vaccine, enrolling NY-ESO-1-expressing tumor or not would be a critical issue to be discussed. Combination vaccines of MAGE-A4 and NY-ESO-1 antigens would be one of the strategies to overcome the poor prognosis.

## INTRODUCTION

The complex of cholesteryl pullulan (CHP) and tumor antigen consists of CHP nanoparticles containing tumor antigen protein, and is a cancer vaccine with a novel antigen delivery system for both MHC class I and class II pathways [1]. Clinical trials have safely and repeatedly administered CHP-HER2 and CHP-NY-ESO-1 vaccines, and both vaccines induced antigen-specific CD8+ and CD4+ T cell immunity as well as humoral immune responses [2–4].

Cancer-testis antigens are expressed in the normal testis and placenta, but may be expressed exclusively in tumor tissues, and as such these antigens are considered to be an ideal target for cancer immunotherapy. Among them, the MAGE-family antigens are frequently expressed in tumor tissues; for example, in esophageal cancer, appoximately 50% of tumors express MAGE-A4, whereas 30% of tumors express NY-ESO-1 [5]. Thus, MAGE-A4 may be an ideal candidate as a target antigen for cancer vaccines.

Although it has been reported that cancer-testis antigens, including the MAGE-family and NY-ESO-1 antigens, were co-expressed on the same tumor tissues [6], the clinical implication of this fact has not been well explored.

We conducted a dose-escalation trial of CHP-MAGE-A4 vaccine with doses of 100 μg and 300 μg in patients with refractory cancers, including esophageal cancer. We evaluated the safety of the vaccine and the immune responses to both MAGE-A4 and NY-ESO-1 antigens. We found that patients with refractory esophageal or head/neck squamous cell carcinomas that co-expressed MAGE-A4 and NY-ESO-1 had a poorer prognosis than those whose tumors expressed MAGE-A4 but not NY-ESO-1. Three patients exhibited immune reactions to a non-vaccine antigen during CHP-MAGE-A4 vaccination.

## RESULTS

### Patient characteristics and clinical safety (Table 1)

**Table 1:** Clinical characteristics of CHP-MAGE-A4 vaccinated cancer patients and their safeties and survivals after vaccinations

Pt No.	Code No.	Age at entry/Sex	Disease	Stage at onset	Prior therapy	Lesions at study entry	Dose (μg)	Vaccine cycle	Related adverse event (grade)	Tumor response (during first six cylcles)	Survival time (month)
1	766	67/M	laryngeal squamous cell cancer	IV	surgery, radiotherapy	skin, bone	100	4	none	PD	2.5
2	630	68/M	esophageal cancer	III	chemotherapy, radiotherapy	lung, liver	100	10	none	PD	7
3	887	82/F	esophageal cancer	IV	radiotherapy	lymph node	100	6	skin reaction(I)	PD	8.5
4	698	48/F	ovarian cancer	IIc	surgery, chemotherapy	none	100	6	skin reaction(I)	PD	100.1^*^
5	687	68/M	esophageal cancer	III	chemotherapy, radiotherapy	esophagus, lymph node	300	4	none	PD	8.8
6	998	62/M	esophageal cancer	IV	chemotherapy, radiotherapy	none	300	15	skin reaction(I)	not evaluable	68.3^*^
7	1147	64/F	esophageal cancer	IV	surgery	lymph node	300	11	skin reaction(I)	SD	10.3
8	1358	38/F	ovarian cancer	IIIc	surgery, chemotherapy	none	300	12	none	not evaluable	87.4^*^
9	1188	60/M	esophageal cancer	III	chemotherapy, radiotherapy	none	300	10	none	not evaluable	84.2^*^
10	704	69/M	esophageal cancer	IV	chemotherapy, radiotherapy	liver	300	6	none	PD	4.1
11	NMC001	76/M	esophageal cancer	II	chemothrapy, radiotherapy	esophagus	300	6	skin reaction(I)	PD	16.3
12	KIT-5	69/M	esophageal cancer	IV	chemotherapy, radiotherapy, immunotherapy	lung, lymph node	100	14	skin reaction(I)	PD	7.5
13	KIT-8	67/M	esophageal cancer	IV	chemotherapy, radiotherapy	primary tumor, lymph node	100	7	skin reaction(I)	PD	3.2
14	KIT-9	56/M	esophageal cancer	IIB	surgery,chemotherapy, radiotherapy	lung, lymph node	100, (−>300)	16	skin reaction(I)	PD	7.6
15	KIT-10	76/F	esophageal cancer	IIIC	chemotherapy, radiotherapy	primary tumor, lymph node	300	7	skin reaction(I)	PD	4.3
16	KIT-11	64/M	esophageal cancer	IV	chemotherapy, radiotherapy	lymph node	300	7	skin reaction(I)	PD	3.4
17	KIT-12	83/M	esophageal and pharyngeal cancer	IVA	chemotherapy, radiotherapy	primary tumor	300	4	none	PD	3.3
18	KIT-13	69/M	duodenal cancer	IIIA	surgery,chemotherapy	lymph node,lung, liver	300	14	skin reaction(I)	PD	8.2
19	KIT-14	72/M	esophageal cancer	IIB	chemotherapy, radiotherapy	primary tumor	300	7	skin reaction(I)	PD	10.9
20	KIT-15	66/M	tongue squamous cell cancer	IVA	surgery,chemotherapy, radiotherapy	pleural dissemination	300+OK432	6	skin reaction(I)	PD	4
21	KIT-16	63/M	parotid cancer	IVA	surgery	pleural dissemination	300+OK432	9	skin reaction(I)	SD	36.6
22	KIT-17	66/M	esophageal cancer	IV	chemotherapy, radiotherapy	lymph node	300+OK432	8	skin reaction(I)	SD	16

^*^alive.

Eleven patients each were enrolled in a Mie University/Nagasaki Medical Center trial and in a Kitano Hospital trial. Sixteen patients had esophageal cancer, 4 had head/neck cancer, 2 had ovarian cancer, and 1 had duodenal cancer (1 patient had both esophageal and pharyngeal squamous cell carcinoma). All enrolled patients had MAGE-A4-positive refractory/advanced or metastatic tumors. Seventeen patients were male, and 5 were female. The patients were aged 38 to 83 years with a median age of 67. The number of vaccination doses administered per patient ranged from 4 to 16, with a median of 7 doses. Fifteen patients developed transient grade 1 skin reactions at the injection sites, which resolved without any treatment. No dose-limiting toxicity was observed. The addition of OK-432 did not increase the rate of adverse events.

### Clinical responses and long-term follow-up (Table 1)

**Figure 1:**
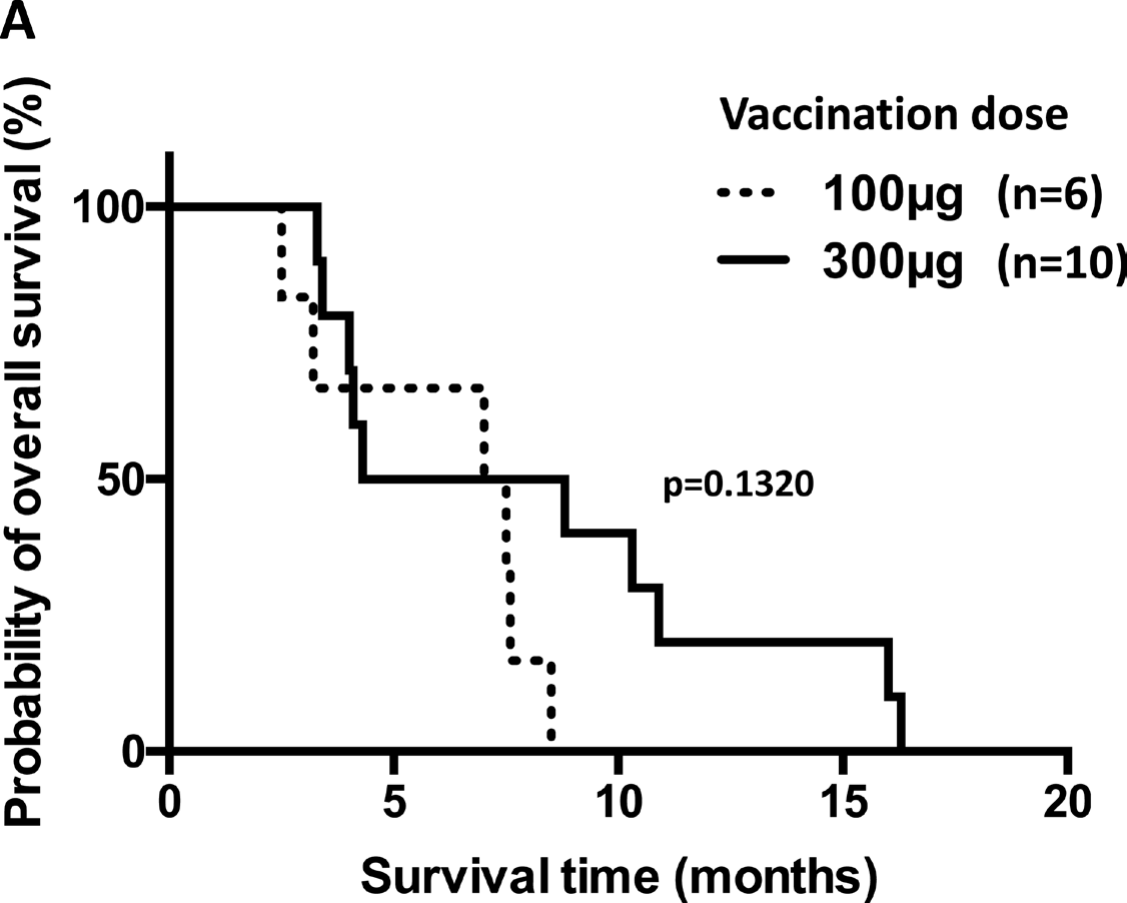

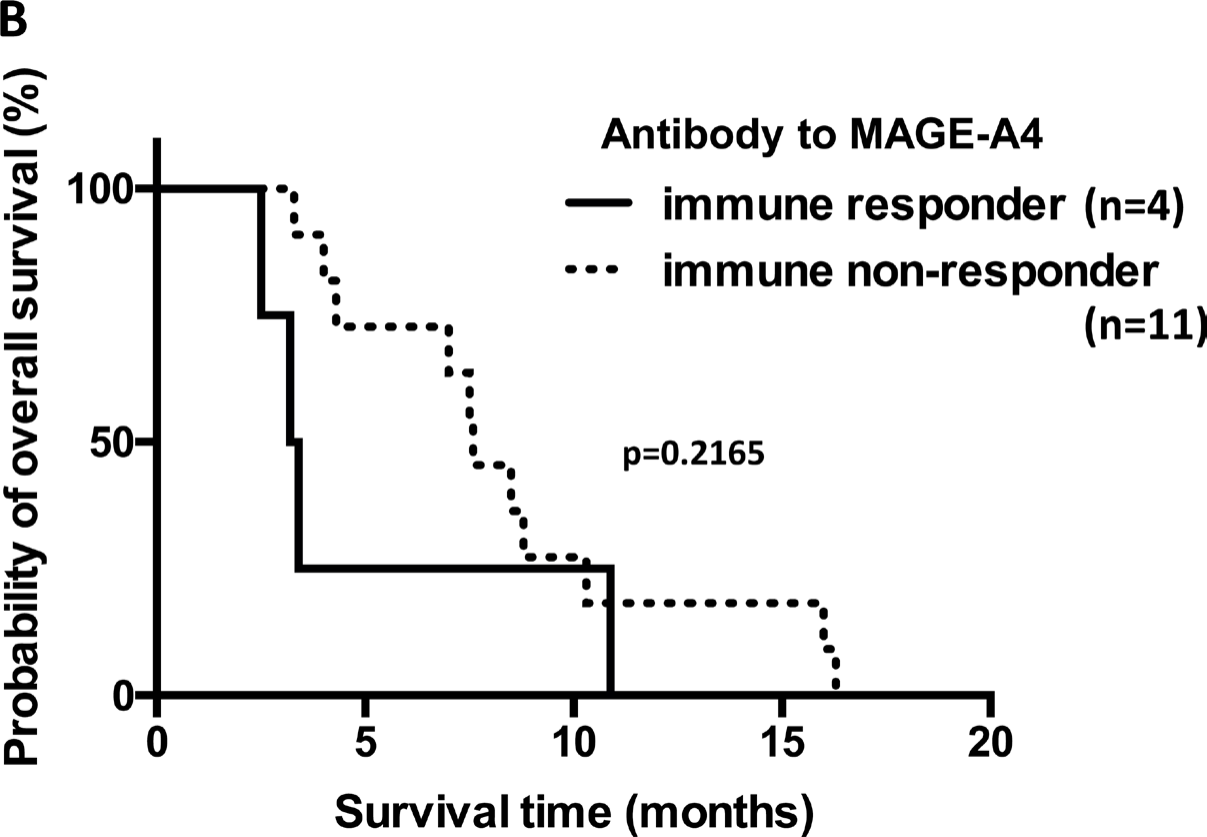
Overall survival of patients with refractory esophageal or head/neck squamous cell carcinoma who received the CHP-MAGE-A4 vaccine (**A**) Kaplan–Meier survival curves of 16 patients with refractory esophageal or head/neck squamous cell carcinoma who were vaccinated with CHP-MAGE-A4. Six patients were given a 100 μg vaccine dose, while the other 10 patients were given the 300 μg dose. The survival times are not statistically different (*p* = 0.1320). (**B**) 15 patients with refractory esophageal or head/neck squamous cell carcinoma, were evaluated for the immune responses to MAGE-A4. Patient No. 10 (code No. 704) was excluded, in whom the antibody datum at post-vaccine was not available. Four patients with esophageal or head/neck squamous carcinoma exhibited immune responses to MAGE-A4. The other 11 patients did not have such responses. The survival times are not statistically different (*p* = 0.2165).

After 6 vaccination cycles, 3 of the 19 evaluable patients showed stable disease (SD), and the other 16 patients showed progressive disease (PD). No patients exhibited tumor regression. The overall survival time was 2.5 to 100.1 months, with a median of 8.4 months. In the cohort receiving the 100 μg dose, survival ranged from 2.5 to 100.1 months, with a median of 7.5 months; in the cohort receiving the 300 μg dose, survival ranged from 4 to 87.4 months, with a median of 10.3 months. The addition of OK-432 did not improve survival. We did not see correlations between the occurrence of skin reactions at the vaccines sites and the clinical responses or survivals. Six patients with esophageal cancer or head/neck cancer who were vaccinated with 100 μg of CHP-MAGE-A4 survived for a median of 7.2 months (range, 2.5 to 8.5). The other 10 patients, who received 300 μg of the vaccine, survived for a median of 6.5 months (range, 3.3 to 16.3) (Figure 1A). These two durations were not significantly different (*p* = 0.1320).

### Expression of NY-ESO-1 antigen in MAGE-A4-expressing tumors (Table 2, Supplementary Table 1)

**Table 2:** Expression of MAGE-A4 and NY-ESO-1 antigens on tumor tissues and humoral immune responses after MAGE-A4 vaccinations

Pt No.	Code No.	MAGE-A4 expression (% in a MCV-1/IHC sample)	Humoral immune response to MAGE-A4				NY-ESO-1 expression (% in a IHC sample)	Humoral immune response to NY-ESO-1			
			Baseline status	OD value (pre)	OD value (post)	Response		Baseline status	OD value (pre)	OD value (post)	Response
1	766	+	−	0.17	0.56	responded	−	+	1.30	1.23	none
2	630	+	−	0.18	0.13	none	+	+	0.67	0.78	none
3	887	+ (40%)	+	1.74	1.73	none	−	−	0.12	0.14	none
4	698	+	−	0.10	0.10	none	−	−	0.09	0.08	none
5	687	+	+	0.60	0.97	none	−	−	0.08	0.10	none
6	998	+	+	0.78	0.64	none	−	+	0.33	0.35	none
7	1147	+	−	0.21	0.31	none	−	−	0.20	0.22	none
8	1358	+	−	0.16	0.28	none	−	−	0.18	0.18	none
9	1188	+	−	0.17	0.17	none	−	−	0.17	0.15	none
10	704	+	−	0.13	NA	NA	+	−	0.08	NA	NA
11	NMC001	+	+	0.65	0.82	none	NA	−	0.14	0.15	none
12	KIT-5	+ (90%)	+	1.91	2.00	none	+ (5%)	+	0.29	1.66	augmented
13	KIT-8	+ (30%)	+	0.65	1.57	augmented	+ (30%)	+	1.71	1.82	none
14	KIT-9	+	−	0.21	0.19	none	−	+	0.32	1.78	augmented
15	KIT-10	+	−	0.08	0.07	none	−	−	0.09	0.07	none
16	KIT-11	+	−	0.16	0.42	responded	NA	−	0.12	0.11	none
17	KIT-12	+	−	0.20	0.20	none	+ (30%)	+	0.69	0.79	none
18	KIT-13	+ (2%^*^)	−	0.26	0.88	responded	+ (70%)	+	1.73	1.78	none
19	KIT-14	+	−	0.09	0.70	responded	−	−	0.07	0.06	none
20	KIT-15	+ (30%)	−	0.23	0.31	none	+ (5%)	+	0.66	1.45	augmented
21	KIT-16	+	−	0.10	0.16	none	−	+	0.27	0.44	none
22	KIT-17	+	−	0.17	0.17	none	−	+	0.36	0.27	none

^*^focally stained.

Cut-off levels for MAGE-A4 and NY-ESO-1 are 0.32 and 0.27, respectively.

Two-fold or more increase from the baseline level in patients with pre-existing antibodies are shown as augmented.

Abbreviations: IHC, immunohistochemistry, NA, not available.

**Figure 2:**
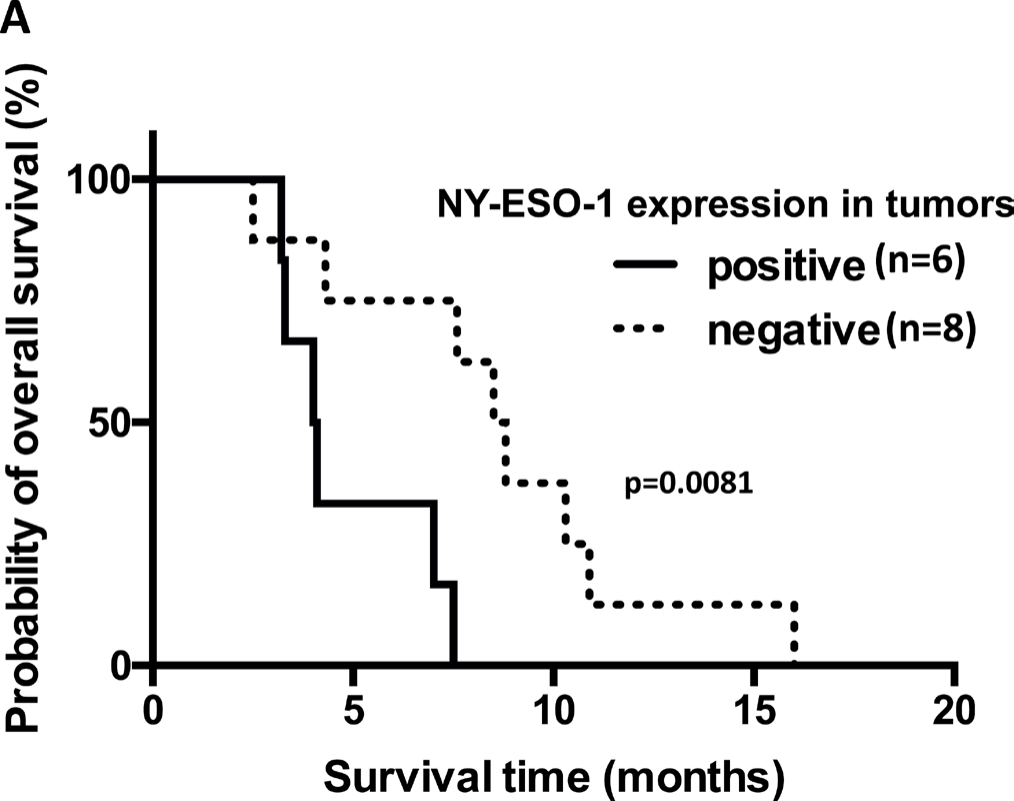

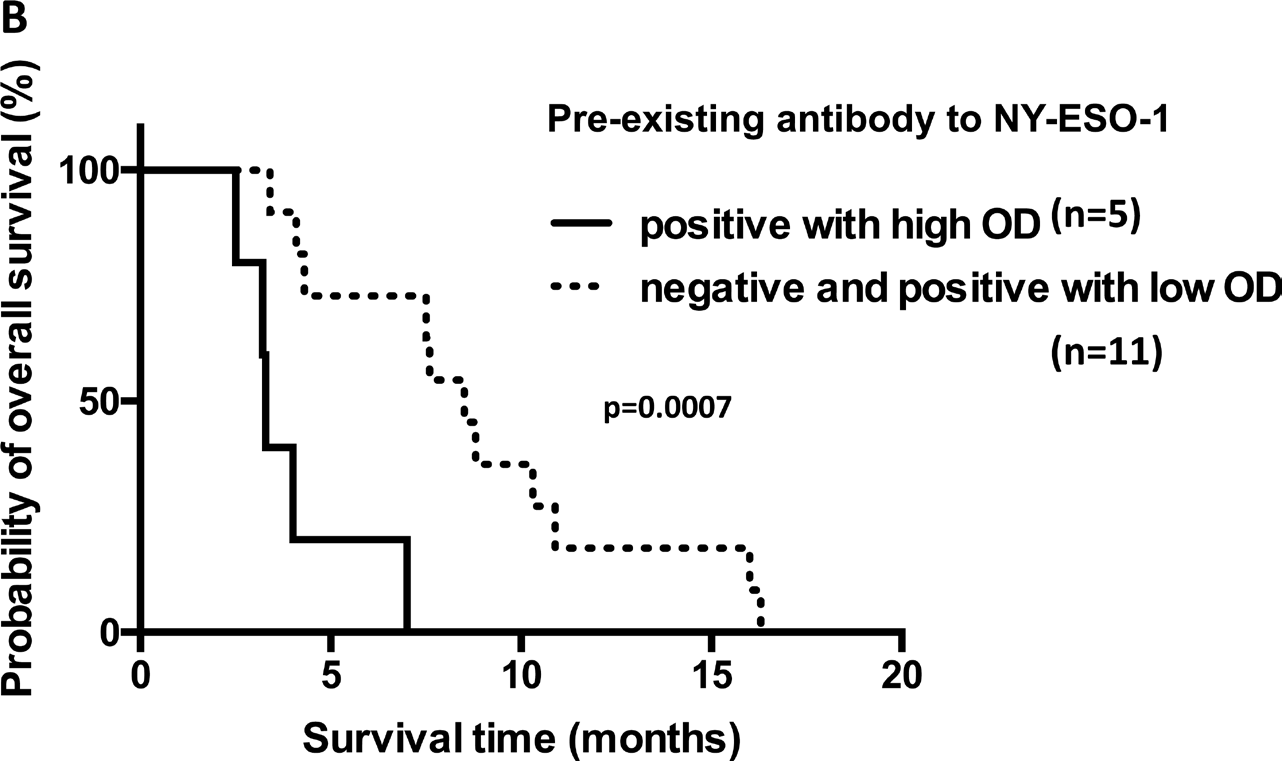
Overall survival of patients with refractory esophageal or head/neck squamous carcinoma who co-expressed NY-ESO-1 or had pre-existing immunity to NY-ESO-1 (**A**) Kaplan–Meier survival curves of 14 patients with refractory esophageal or head/neck squamous cell carcinoma who received the CHP-MAGE-A4 vaccine. Six patients had NY-ESO-1-expressing tumors and 8 had NY-ESO-1-negative tumors. Survival time was significantly longer in esophageal or head/neck squamous carcinoma patients with NY-ESO-1-negative tumors than in those with NY-ESO-1-positive tumors (*p* = 0.0081). (**B**) Overall survival of patients with or without pre-existing anti-NY-ESO-1 antibody. Survival time was significantly shorter in esophageal or head/neck squamous carcinoma patients with high levels of pre-existing antibody than those without it, including those with low titers of the antibody (*p* = 0.0007).

Of the 22 patients with MAGE-A4-expressing tumors, 7 had tumors that co-expressed the NY-ESO-1 antigen. Five of these 7 patients had esophageal carcinomas and 1 each had a duodenal carcinoma and tongue carcinoma. Among the 22 patients, 16 patients had refractory esophageal or head/neck squamous cell carcinoma. Fourteen were assessed for NY-ESO-1 antigen expression using tumor samples. (Supplementary Figure 1). Six had NY-ESO-1-expressing tumors and 8 had NY-ESO-1-negative tumors; their median survival times were 4.0 (range, 3.2 to 7.5) and 8.6 months (range, 2.5 to 16.0), respectively. The survival time was significantly longer in the patients with NY-ESO-1-negative tumors (*p* = 0.0081) (Figure 2A).

### Immune responses to MAGE-A4 after CHP-MAGE-A4 vaccinations (Table 2, Table 3)

**Table 3:** Immune responses to MAGE-A4 and NY-ESO-1 in 21 patients vaccinated with CHP-MAGE-A4

	Number of patients	Immune responder to MAGE-A4	Response rate (%)
negatives of pre-existing antibody to MAGE-A4	15^*^	4	27
positives of pre-existing antibody to MAGE-A4	6	1	17
vaccine of 100 μg dose	7	2	29
vaccine of 300 μg dose	14^*^	3	21

	Number of patients	Immune responder to NY-ESO-1	Response rate (%)
positives of pre-existing antibody to NY-ESO-1	11	3	27
negatives of pre-existing antibody to NY-ESO-1	10^*^	0	0

^*^patient No. 10 (code No. 704) was excluded, in whom the antibody data at post-vaccine was not available.

Six of the 22 (27%) patients had pre-existing antibodies to MAGE-A4, including 5 with high optical density (OD) (at least twice the cut-off level) and 1 with low OD (below twice the cut-off level) value.

To evaluate the antibody responses after vaccination, serum samples collected at each vaccination were analyzed using a MAGE-A4-specific IgG ELISA. Of the 15 patients who were antibody-negative before vaccination, 4 (27%) became seropositive. One of 6 (17%) initially antibody-positive patients had augmented antibody responses. In total, 5 of 21 (24%) patients exhibited immune responses. In the 7 patients receiving the 100 μg dose, 2 (29%) showed an immune response, whereas 3 of the 14 (21%) patients receiving the 300 μg dose exhibited a response. The 4 patients with esophageal or head/neck squamous cell carcinoma who exhibited an immune response to MAGE-A4 survived for a median of 3.3 months (range, 2.5 to 10.9). The other 11 patients with no immune response survived for a median of 7.6 months (range, 3.3 to 16.3) (Figure 1B). These survival times were not significantly different (*p* = 0.2165).

### Spreading immune response to NY-ESO-1 after CHP-MAGE-A4 vaccinations

As shown in Tables 2 and 3, 11 of the 22 (50%) patients had pre-existing antibodies to NY-ESO-1, including 6 with high OD values. In 7 patients whose tumors expressed both MAGE-A4 and NY-ESO-1 antigens, 6 (86%) had pre-existing antibodies to NY-ESO-1.

**Figure 3:**
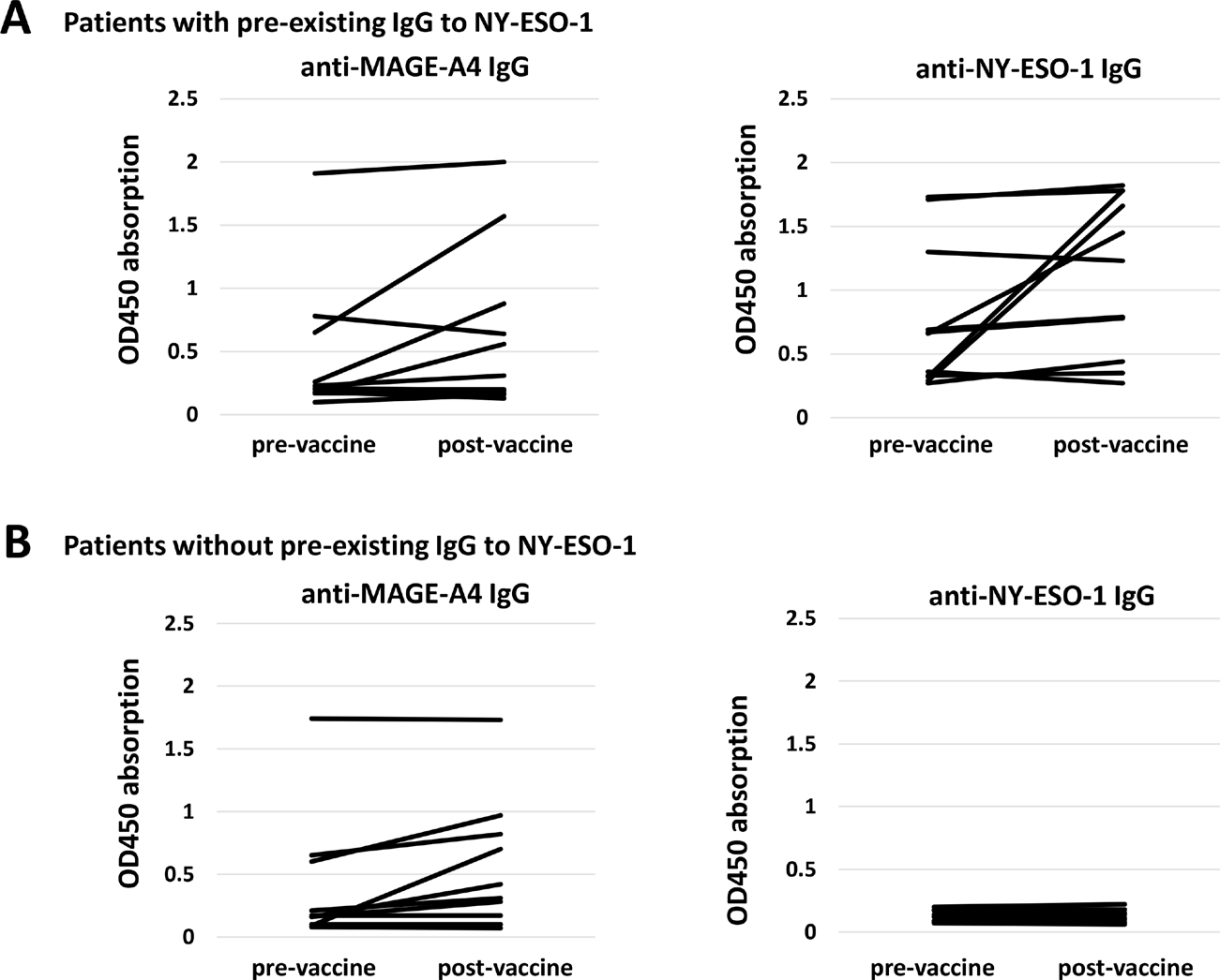

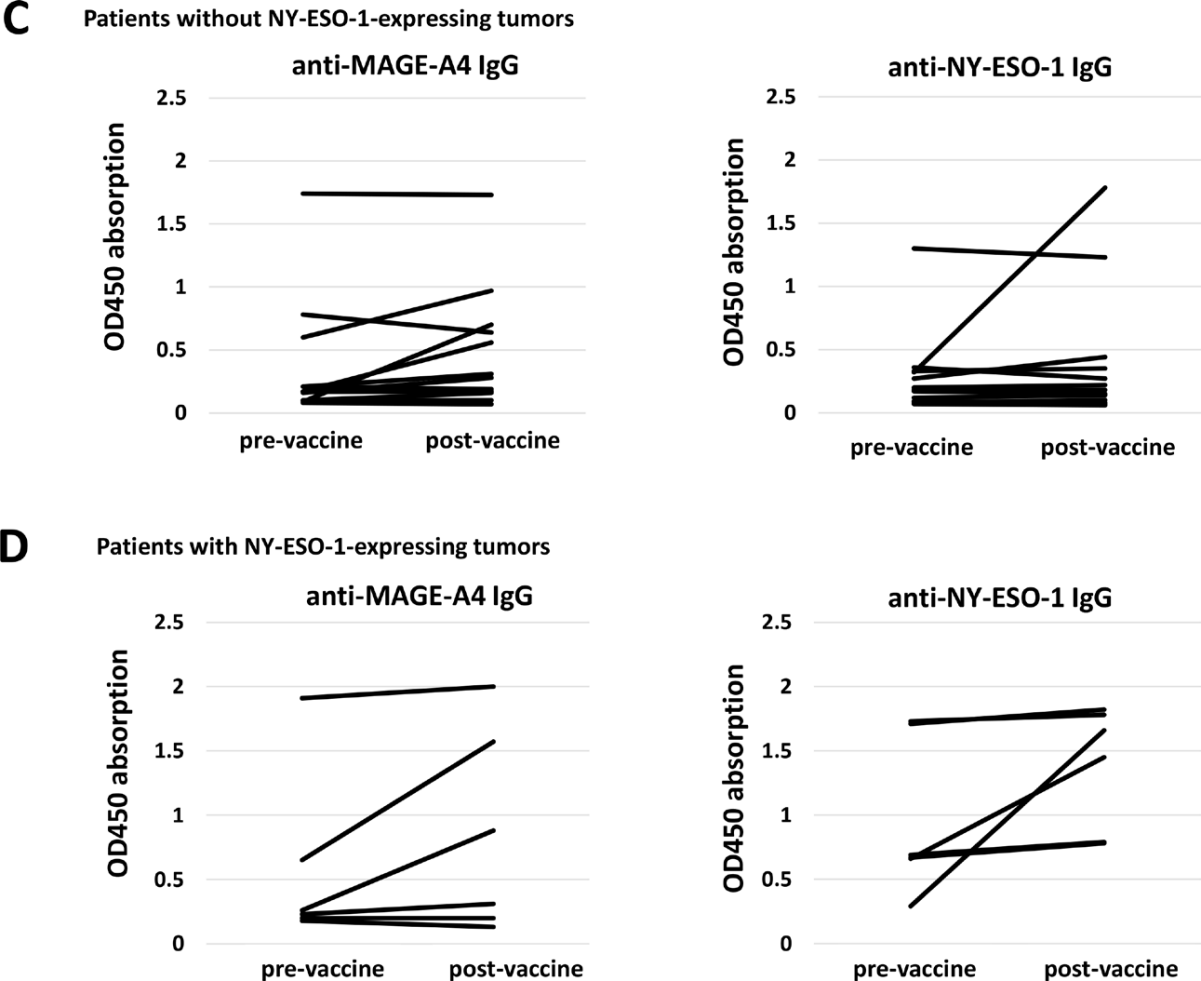
Antibody responses after CHP-MAGE-A4 vaccinations (**A**) Left panel shows IgG responses measured by ELISA assay to MAGE-A4 protein in patients who had pre-existing IgG to NY-ESO-1 antigen (*n* = 11). Right panel shows IgG responses to NY-ESO-1 protein in the same patients. (**B**) Left panel shows IgG responses measured by ELISA assay to MAGE-A4 protein in patients who did not have pre-existing IgG to NY-ESO-1 antigen (*n* = 10). Right panel shows IgG responses to NY-ESO-1 protein in the same patients. (**C**) Left panel shows IgG responses to MAGE-A4 protein in patients whose tumors did not express NY-ESO-1 antigen (*n* = 13). Right panel shows IgG responses to NY-ESO-1 protein in the same patients. (**D**) Left panel shows IgG responses to MAGE-A4 protein in patients whose tumors expressed NY-ESO-1 antigen (*n* = 6). Right panel shows IgG responses to NY-ESO-1 protein in the same patients.

**Figure 4:**
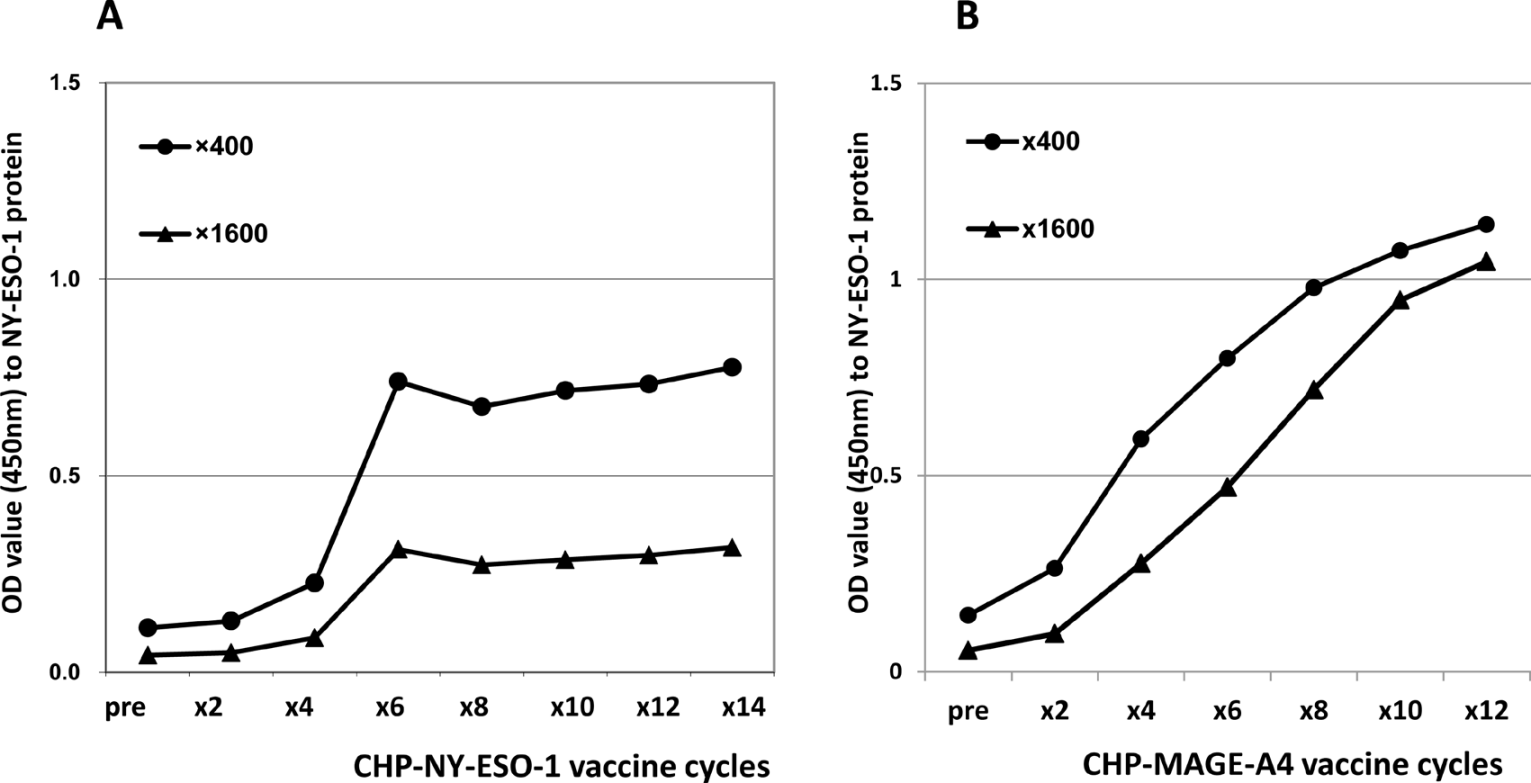
Antibody responses to NY-ESO-1 in a patient who received prior vaccine with NY-ESO-1 protein and the present vaccine with MAGE-A4 (**A**) IgG responses measured by ELISA assay to NY-ESO-1 protein in patient No.12 (KIT-5). The serum was diluted by 400 or 1,600 and assayed by ELISA. No antibody response existed before the vaccination. Six cycles of CHP-NY-ESO-1 vaccine at 100 μg per dose induced antibody responses. The intensities plateaued following the repeated vaccinations. (**B**) IgG responses to NY-ESO-1 protein. The NY-ESO-1-antibody response disappeared before vaccination. Four cycles of CHP-MAGE-A4 vaccine at 100 μg per dose induced antibody responses to NY-ESO-1. The intensities increased with repeated vaccinations.

Of the 21 patients overall, 3 exhibited immune responses to NY-ESO-1 during CHP-MAGE-A4 vaccination, and all 3 of these patients had pre-existing antibody responses to NY-ESO-1. Eleven patients were initially seropositive for NY-ESO-1, and 27% (3/11) of these showed an immune response to this antigen (Tables 2 and 3, Figure 3A). The 3 patients (Nos. 12, 14, and 20) who developed spreading immune responses to NY-ESO-1 did not exhibit an immune response to MAGE-A4 (Table 2, Figure 3A). In contrast, none of the patients without pre-existing immunity to NY-ESO-1 showed an immune response to NY-ESO-1 (Table 2, Figure 3B). Of the 13 patients whose tumors did not express the NY-ESO-1 antigen, 1 patient showed an antibody response to NY-ESO-1 (Table 2, Figure 3C). Of 7 patients whose tumors expressed both antigens, 2 showed antibody responses to NY-ESO-1 (Table 2, Figure 3D). One esophageal cancer patient, No. 12 or code No. KIT-5, was vaccinated with NY-ESO-1 protein in a prior clinical trial [4]. At the beginning of that trial the patient did not have pre-existing antibodies to the NY-ESO-1 antigen, and the response was induced over the course of the vaccinations (Figure 4A). Twelve months after the CHP-NY-ESO-1 vaccinations, the patient’s antibody level had decreased to a marginal level of 0.29 OD (Table 2, Figure 4B). The patient did have pre-existing antibodies (1.91 OD) to MAGE-A4 antigen at the start of the current trial, and received 14 cycles of the CHP-MAGE-A4 vaccine. Interestingly, no augmentation of immunity to MAGE-A4 was seen, and instead, a definite antibody response to NY-ESO-1 was observed over the course of the CHP-MAGE-A4 vaccinations (Figure 4B).

The median survival time in the 5 patients with high levels of pre-existing NY-ESO-1 antibodies was 3.3 months (range, 2.5 to 7.0), while the 11 patients in whom these antibodies were either absent or occurred at low levels survived a median of 8.5 months (range, 3.4 to 16.3). Survival time was significantly longer in NY-ESO-1 seronegative patients, including those with low titers of the antibody, than in those with high levels of pre-existing NY-ESO-1 antibodies (*p* = 0.0007) (Supplementary Figure 1, Figure 2B).

### Seromics: array profiling assay

**Figure 5:**
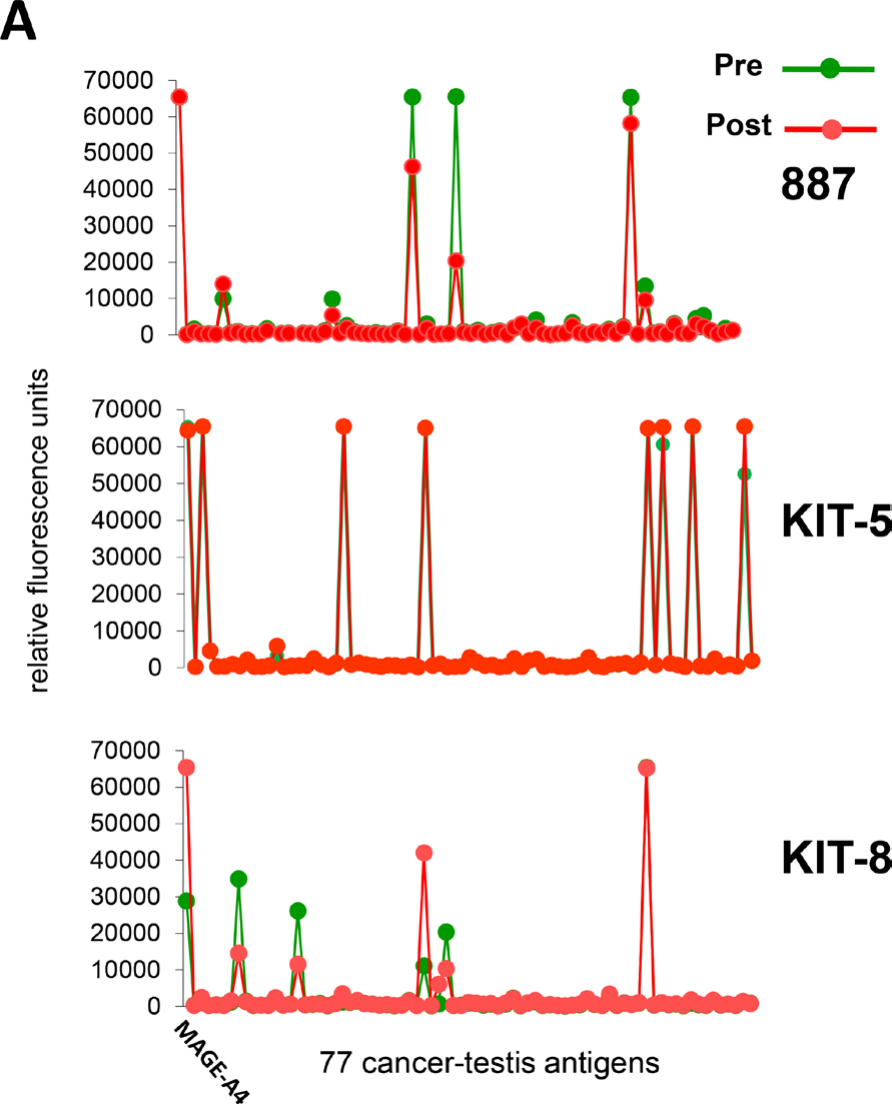

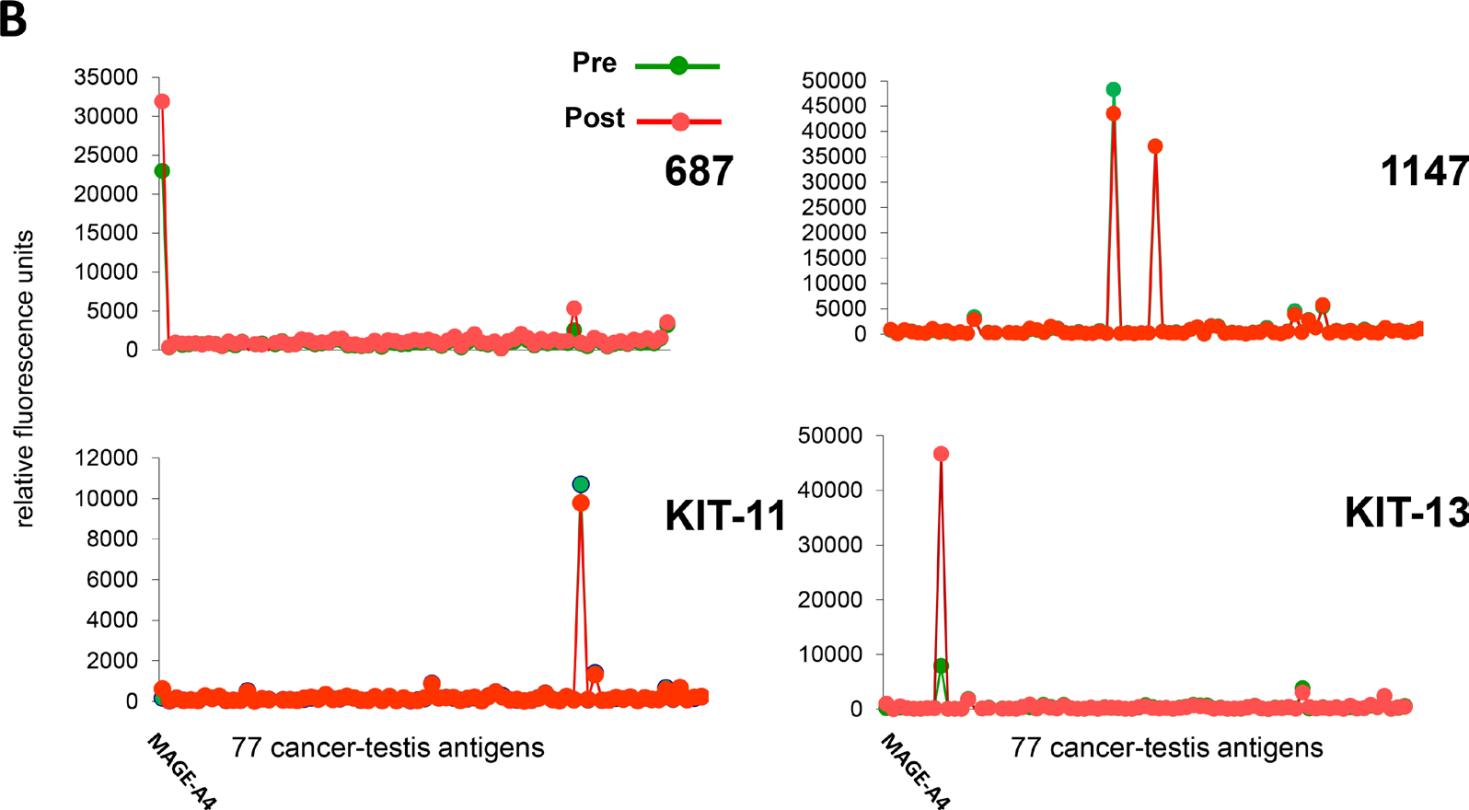
Array profiling assay ProtoArray was performed using 1:500 diluted serum from 7 patients who received 4 or more vaccinations with CHP-MAGE-A4. The X-axis shows 77 cancer-testis antigens, including MAGE-A4. Responses occurred to the same antigens in pre-vaccine and post-vaccine sera, with varying intensities. In the X-axis, the 77 antigens were listed in the same order as those in Table 4, from the top of the left column to the bottom of the right column. (**A**) Three patients, 887, KIT-5, and KIT-8, were vaccinated with 100 μg of CHP-MAGE-A4. (**B**) Four patients, 687, KIT-11, 1147, and KIT-13, were vaccinated with 300 μg of CHP-MAGE-A4.

Seven patients provided serum samples both before and after vaccine administration, and sera were assayed using ProtoArray microarrays. In Figure 5A and 5B, the response patterns to 77 cancer-testis antigens (Table 4) are shown for both time periods. While different patients responded to different antigens, each individual patient responded to the same antigens both before and after vaccination. This indicates that patients had antibodies to multiple antigens prior to CHP-MAGE-A4 vaccinations and that spreading immune reactions arose from these pre-existing responses.

**Table 4:** 77 selected cancer-testis antigens that are reacted to sera from pre- and post-vaccine timings

melanoma antigen family A, 4 (MAGEA4), transcript variant 2	synovial sarcoma, X breakpoint 4, mRNA (cDNA clone MGC:119056 IMAGE:40003338), complete cds
acrosin binding protein (ACRBP)	chromosome X open reading frame 48 (CXorf48)
melanoma-associated antigen 2	family with sequence similarity 46, member D (FAM46D)
ankyrin repeat domain 45 (ANKRD45)	transmembrane phosphatase with tensin homology (TPTE)
transmembrane protein with EGF-like and two follistatin-like domains 1 (TMEFF1)	coiled-coil domain containing 33 (CCDC33), transcript variant 1
cell differentiation protein RCD1 homolog	SPANX family, member C (SPANXC)
melanoma antigen family B, 2 (MAGEB2)	P antigen family, member 2 (prostate associated) (PAGE2)
PDZ binding kinase (PBK)	SPANX family, member E (SPANXE)
DEAD (Asp-Glu-Ala-Asp) box polypeptide 43 (DDX43)	outer dense fiber of sperm tails 2 (ODF2), transcript variant 1
PAS domain containing 1 (PASD1)	testis specific, 10 (TSGA10)
synaptonemal complex central element protein 1 (SYCE1)	SPANX family, member N3 (SPANXN3)
B melanoma antigen 3	testis expressed 101 (TEX101)
maelstrom homolog (Drosophila) (MAEL)	P antigen family, member 5 (prostate associated) (PAGE5)
outer dense fiber of sperm tails 3 (ODF3)	zinc finger protein 165 (ZNF165)
G patch domain containing 2 (GPATCH2)	chromosome X open reading frame 48 (CXorf48), transcript variant 2
PDZ binding kinase (PBK)	outer dense fiber of sperm tails 2 (ODF2)
G antigen 1 (GAGE1)	POTE ankyrin domain family, member B, mRNA (cDNA clone MGC:119373 IMAGE:40006489), complete cds
Melanoma-associated antigen B3	Down syndrome critical region protein 8
heat shock protein, alpha-crystallin-related, B9 (HSPB9)	cytochrome c oxidase subunit VIb polypeptide 2 (testis) (COX6B2)
ADAM metallopeptidase domain 2 (fertilin beta) (ADAM2)	nucleolar protein 4, mRNA (cDNA clone MGC:8430 IMAGE:2821116), complete cds
cancer/testis antigen family 45, member A1 (CT45A1), mRNA.	synaptonemal complex central element protein 1 (SYCE1), transcript variant 2
melanoma antigen family A, 12 (MAGEA12)	P antigen family, member 1 (prostate associated) (PAGE1)
testis-specific serine kinase 6 (TSSK6)	Dual specificity protein kinase TTK
chondrosarcoma associated gene 1 (CSAG1)	Melanoma-associated antigen 3
LEM domain-containing protein 1	nuclear RNA export factor 2 (NXF2), transcript variant 1
lactate dehydrogenase C (LDHC), transcript variant 1	melanoma antigen family B, 4 (MAGEB4)
TTK protein kinase (TTK)	Protein FAM133A
LEM domain-containing protein 1	CPX chromosome region, candidate 1 (CPXCR1)
Sperm surface protein Sp17	spermatogenesis associated 19 (SPATA19)
preferentially expressed antigen in melanoma (PRAME)	Melanoma-associated antigen 2
Sperm protein associated with the nucleus on the X chromosome N4	synovial sarcoma, X breakpoint 5 (SSX5)
calreticulin 3 (CALR3)	sperm associated antigen 9 (SPAG9)
melanoma antigen family A, 10 (MAGEA10), transcript variant 2	synovial sarcoma, X breakpoint 3 (SSX3), transcript variant 1
P antigen family, member 5 (prostate associated) (PAGE5), transcript variant 1	tubby like protein 2 (TULP2)
X antigen family, member 2 (XAGE2)	TTK protein kinase (TTK)
PDZ binding kinase (PBK)	interleukin 13 receptor, alpha 2 (IL13RA2)
centrosomal protein 290kDa (CEP290)	melanoma antigen family B, 1 (MAGEB1), transcript variant 1
outer dense fiber of sperm tails 4 (ODF4)	Sperm protein associated with the nucleus on the X chromosome D
SPANX family, member B1 (SPANXB1)	

77 antigens were listed in the same order as Figure 5, from the left to the right in the X-axis.

## DISCUSSION

In this phase I clinical trial, we evaluated the safety and immunogenicity of the CHP-MAGE-A4 cancer vaccine, and found that the vaccine was safe and that immune responses were induced in 24% of 21 patients with advanced cancer. The 100 μg vaccine dose resulted in immune responses in 29% of patients, compared with 21% for the 300 μg dose, indicating no dose-dependency. We previously reported that the NY-ESO-1 protein vaccine complexed with CHP showed dose-dependent immune responses [7]. Regarding the current CHP-MAGE-A4 trial, it is unclear if the low rate of immune responses indicates that a higher dose of MAGE-A4 should be used in the vaccine, or if the MAGE-A4 protein is not sufficiently immunogenic. A future phase II dose-escalation trial should be conducted to determine a recommended dose.

Of the 22 patients in this study, 27% had pre-existing antibody responses to the MAGE-A4 antigen. This rate is similar to other cancer-testis antigens, such as NY-ESO-1, and indicates that the MAGE-A4 antigen is immunogenic enough to induce immune reactions in hosts bearing MAGE-A4-expressing tumors [8]. We also found that 7 of 20 (35%) cases of MAGE-A4-expressing tumors simultaneously expressed NY-ESO-1. Six of the 7 (86%) patients who expressed both antigens had pre-existing immunity to NY-ESO-1 as well. This indicates that expression of multiple cancer-testis antigens might create high immunogenic activity in cancer-bearing hosts.

We observed antigen spreading to NY-ESO-1 during CHP-MAGE-A4 vaccinations in three (14%) patients, all of whom had pre-existing NY-ESO-1 immunity. In contrast, no spreading reactions were induced in patients who did not have these pre-existing antibodies. To examine if pre-existing immunity is required for spreading immune responses, we used ProtoArray microarrays to analyze patient sera obtained before and after vaccine administration. As shown in Figure 5, CHP-MAGE-A4 vaccination induced the response against pre-existing antigen rather than *de novo* immune response. CHP and/or OK-432 may contribute to augment the subliminal response. Also, it is possible that some of these proteins in Table 4 may attenuate immune regulation in tumor micro-environment. To confirm the mechanism of antigen spreading, accumulation of more data would be necessary. In a previous report that investigated antigen spreading in CHP-NY-ESO-1-vaccinated patients, 8 of 9 patients with immune responses to NY-ESO-1 also responded to antigens other than NY-ESO-1, including MAGE-A4 [9]. Since these patients had tumors that expressed one or more antigens other than NY-ESO-1, we can conclude that antigen spreading may often occur over the course of vaccination in patients whose tumors express multiple antigens.

The clinical significance of such spreading immune reactions has not been well investigated. However, one study demonstrated that T cells that recognized a non-vaccine antigen, a neo-antigen in this case, was a primary contributor to tumor regression in a MAGE-vaccinated melanoma patient [10]. In the current trial, of the 16 patients with either refractory esophageal cancer (*n* = 14) or head/neck squamous cell carcinoma (*n* = 2), the 3 patients who demonstrated antigen spreading survived for a median of 7.5 months, while the 12 patients without spreading survived for 7.7 months. These times were similar. Notably, one patient, KIT-5, had a long survival for more than 3 years from the onset of esophageal cancer, and marked immune spreading to NY-ESO-1 was induced during CHP-MAGE-A4 vaccination. Given these findings, further studies should investigate to clarify the association of antigen spreading and survival impacts in vaccinated esophageal cancer patients.

It has been reported that NY-ESO-1 antibody may be related to tumor burden in patients with NY-ESO-1-expressing tumors. One study found that 31% of esophageal cancer patients had NY-ESO-1 auto-antibodies, and the positive rate increased with tumor stage progression [8]. Another study demonstrated that NY-ESO-1 antibody positivity increased with disease progression in gastric cancer patients, and that antibody levels decreased with surgical resection and chemotherapies [11]. The spreading reaction to NY-ESO-1 in the 3 esophageal cancer patients in this study could be explained by increasing tumor burden, as all 3 patients had distant metastases at study entry and all showed tumor progression after vaccination.

For the first time, we demonstrated that patients with refractory esophageal or head/neck squamous cell carcinoma that co-expressed MAGE-A4 and NY-ESO-1 had significantly worse prognosis than patients with tumors expressing MAGE-A4 alone. Previous studies in several cancers found that NY-ESO-1 expression had varying effects on prognosis. With regard to esophageal cancer, one study suggested that NY-ESO-1 expression was associated with favorable overall survival [12]. Akcakanat *et al.* investigated 213 esophageal cancer patients and demonstrated no impact of NY-ESO-1 expression on either progression-free survival or overall survival [6]. The latter study enrolled 111 MAGE-A-expressing esophageal cancer patients, of whom 32 (28.8%) co-expressed NY-ESO-1, and found that MAGE-A and NY-ESO-1 co-expression did not affect prognosis. These results are inconsistent with our findings and might reflect differences in the clinical status of the investigated cohorts; our study included refractory tumors, while the study by Akcakanat *et al.* investigated newly diagnosed cases. A large study that examined ovarian tumor samples from 1,002 patients showed significantly worse clinical outcomes in patients whose tumor tissues expressed NY-ESO-1 [13], and found that if these patients enrolled in cancer vaccine trials, their overall survival was prolonged. In our trial, despite the CHP-MAGE-A4 vaccine, patients with NY-ESO-1-expressing tumors had significantly shorter survival than those without NY-ESO-1 expression. One explanation would be an adverse influence of NY-ESO-1 antigen expression in tumors, and it is not known if NY-ESO-1 expression is related the disease aggressiveness, or it is associated with disease progression. Expression of MAGE-A4 has not been known to be related to disease prognosis. However, in our study the biological role of co-expression of the two antigens is still unknown. Thus, the MAGE-A4 vaccine should not have been administered to the group with NY-ESO-1 expression, and instead a NY-ESO-1 vaccine might be chosen to mitigate the worse prognosis. The other strategy would be combination vaccine of MAGE-A4 and NY-ESO-1 antigens, which could overcome the worse prognosis of the two-antigen expressing esophageal or head/neck cancer patients in the future clinical trial.

Two other independently conducted clinical trials of the CHP-MAGE-A4 vaccine have been reported to date [14, 15]. Saito *et al.* reported that 4 of 20 (20%) patients receiving a 300 μg dose developed antibody responses, and these patients had significantly longer survival than the patients with no immune responses. In contrast, in our study we saw no difference in survival between patients with and without immune responses. Other factors may have influenced the clinical outcomes in our study population, although our rate of immune reaction was slightly higher. Miyauchi *et al.* reported that 6 patients (50%) developed spreading immune reactions to NY-ESO-1 and that the spread to the other antigens had no relation to clinical outcomes. Their findings are compatible with our own.

In conclusion, the CHP-MAGE-A4 vaccine was well tolerated in patients with refractory cancer, 24% of whom exhibited immune responses to MAGE-A4 following the 100 μg or 300 μg vaccine doses. Also, we found that patients with esophageal or head/neck squamous cell carcinoma often demonstrated co-expression of MAGE-A4 and NY-ESO-1 in their tumors, and this was associated with worse prognosis, especially in those who had pre-existing antibodies to NY-ESO-1. Antigen spreading occurred in patients who had already been sensitized with primary reactions to other antigens when they were vaccinated with MAGE-A4 cancer vaccine. Therefore, in planning clinical trials of MAGE-A4 vaccine, enrolling NY-ESO-1-expressing tumor or not would be a critical issue to be discussed. Combination vaccines of MAGE-A4 and NY-ESO-1 antigens would be one of the strategies to overcome the poor prognosis.

## PATIENTS AND METHODS

### Preparation of the CHP-MAGE-A4 complex vaccine

The CHP-MAGE-A4 complex vaccine was provided by ImmunoFrontier, Inc. (Tokyo, Japan). Full-length MAGE-A4 cDNA was cloned into the pET vector and introduced into *Escherichia coli* cells. The produced protein was recovered and highly purified using a combination of chromatographic techniques, including metal-chelating affinity chromatography, anion exchange chromatography, size exclusion chromatography, and hydroxyapatite chromatography. CHP was synthesized by a chemical reaction between pullulan (average molecular weight: 100 kDa) and cholesterol isocyanate in pyridine/dimethyl sulfoxide solution (NOF Corporation, Tokyo, Japan). After purification by extraction and precipitation, the resultant CHP was emulsified in water, then freeze dried. When dissolved in water or buffers, CHP spontaneously forms nanoparticles (20–50 nm). The hydrophobic domains of cholesterol on the inside of these nanoparticles associate with the hydrophobic regions of the MAGE-A4 protein, forming a stable complex in solution. This complex of protein and CHP was used as the CHP-MAGE-A4 vaccine. All processes were performed following current Good Manufacturing Practices. The toxicity of the drug product was assessed using animal models, and stability was monitored during the clinical trial using representative samples of the investigational drug product.

### Study design

Two phase I open-label clinical trials were conducted independently, one at Mie University Hospital and Nagasaki Medical Center, and the other at Kitano Hospital. Eleven patients, each with refractory tumor expressing MAGE-A4, were enrolled in each trial: patient numbers 1–11 and 12–22, respectively (Table 1). The results were analyzed together, because these 2 clinical trials were conducted using the same vaccine for the same assessment of MAGE-A4 tumor expression. Primary objectives of each study were safety, specifically maximum tolerable dose, dose-limiting toxicity, and profiles of adverse events, and efficacy, specifically MAGE-A4-specific immune responses. The secondary objective of each study was clinical efficacy, specifically tumor responses and overall survival.

Patients were eligible for entry if they had a performance status (PS) of 0, 1, or 2, were at least 20 years old, had a life expectancy of 4 months or more, and did not have impaired organ function. Patients were ineligible if they were positive for HIV antibody; had multiple active cancers, autoimmune disease, serious allergic history, or active brain metastasis; or received systemic steroids or immunosuppressive therapy within 4 weeks prior to the start of this study.

Three patients in each trial were given 100 μg of CHP-MAGE-A4 every 2 weeks. If there were no adverse events greater than grade 2, the next 3 patients in each trial were immunized with 300 μg of CHP-MAGE-A4 every 2 weeks. If there were no adverse events greater than grade 2, more patients were enrolled into the 300 μg CHP-MAGE-A4 cohort in the trial at Mie University and Nagasaki Medical Center, and into the 300 μg CHP-MAGE-A4 plus immunoadjuvant, OK-432 (Chugai Pharmaceutical Co Ltd, Tokyo, Japan) cohort in the trial at Kitano Hospital. OK-432 is a penicillin-killed and lyophilized preparation of a low-virulence strain of Streptococcus pyogenes (group A). It works as an immune-modulator, and was reported to stimulate toll-like receptor (TLR)-4 and to activate antigen-presenting cells [16]. Clinical responses were assessed according to the Response Evaluation Criteria in Solid Tumors (RECIST) ver 1.1. Each patient received 6 doses of the CHP-MAGE-A4 vaccine. Patients could receive additional treatments if they wished, as long as they had a PS of 2 or less. Safety was evaluated according to the National Cancer Institute Common Terminology Criteria for Adverse Events (CTCAE) ver 3.0. All the safety information was collected and evaluated, and the dose escalation was judged by the independent Data and Safety Committee.

The study was performed in accordance with the current version of the Declaration of Helsinki. Each protocol was approved by the Human Ethics Committee at each hospital. Written informed consent was obtained from each patient enrolled in the trials. Clinical trials conducted at Mie University Hospital/Nagasaki Medical Center and Kitano Hospital were registered in the UMIN Clinical Trials Registry as UMIN000001599 and UMIN000002153, started on December 25, 2008 and July 2, 2009, and ended on November 23, 2012 and July 31, 2014, respectively.

### Expression of MAGE-A4 and NY-ESO-1 antigen in tumor tissues

MAGE-A4 expression was assessed by immunohistochemistry (IHC) using the monoclonal antibodies 57B, MCV-1, and MCV-4, as previously described [17], or by quantitative real-time PCR (qRT-PCR) using specific primers [18]. NY-ESO-1 expression was assessed by immunohistochemistry with the monoclonal antibody E978 (Sigma-Aldrich, Saint Louis, MO) [19], or quantitative qRT-PCR using specific primers [18]. Tissue samples with a 5% or higher positively stained area were judged as antigen positive. Focally stained samples were also positive. Tumor samples expressing 12.2 or more PCR-amplified copies were judged as MAGE-A4 positive. Tumor samples also expressing 1.0 or more PCR-amplified copies were judged as NY-ESO-1 positive.

### Serum samples

To analyze antigen-specific antibody responses, sera were collected at baseline and at 2 weeks after each vaccination. All sera were stored at –80° C until analysis.

### Antibody responses to MAGE-A4 and NY-ESO-1 antigens

Specific antibodies to MAGE-A4 and NY-ESO-1 antigens in the sera were measured by ELISA as described previously [20]. Briefly, recombinant MAGE-A4 and NY-ESO-1 proteins (GST-tag) were absorbed onto immunoplates (442404; Nunc, Roskilde, Denmark) at a concentration of 10 ng/50 µL/well at 4° C. The collected serum samples were diluted from 1:400 to 4 times dilution. After washing and blocking the plate, the sera were added and incubated for 10 h. After washing, goat anti-human IgG (H+L chain) (MBL, Nagoya, Japan) conjugated with peroxidase (The Binding Site, San Diego, CA) was added. After addition of tetramethyl benzidine substrate (Pierce, Rockford, IL), the plate was read using a Microplate Reader (model 550; Bio-Rad, Hercules, CA).

Serum samples from healthy volunteers were evaluated to determine a cut-off level for the anti-MAGE-A4 and anti-NY-ESO-1 antibodies based on the OD_450–550_ absorption value. Serum samples from 20 healthy volunteers were obtained, and assayed in ELISA for MAGE-A4 and NY-ESO-1 IgG antibodies. The cut-off level for each IgG was defined as each mean OD_450–550_ absorption value + 1.65 × standard deviation (SD) value. The cut-off level of anti-MAGE-A4 and anti-NY-ESO-1 IgG were 0.32 and 0.27, respectively. A sample was considered to be positive for anti-MAGE-A4 and anti-NY-ESO-1 antibodies if the optical density (OD)_450–550_ absorption value on ELISA was at the cut-off level or higher at a serum dilution of 1:400. The immune responses of patients with pre-existing anti-MAGE-A4 or anti-NY-ESO-1 antibodies were judged as showing augmentation if the OD values increased by 2-fold or greater.

### Seromics: array profiling assay

ProtoArray microarrays (v4.0; Invitrogen) were purchased and used according to the manufacturer’s instructions. Briefly, after blocking for 1 h at 4° C and washing, arrays were incubated in Quadriperm dishes (Greiner Bio One) placed on a horizontal shaker (50 rpm) for 90 min at 4° C with individual sera diluted 1:500 in 5 ml washing buffer (0.1% Tween 20 [vol/vol], 1% BSA [wt/vol] in PBS). After washing, binding of IgG was detected by incubation with Alexa Fluor 647 goat anti-human IgG (Invitrogen) diluted 1:2,000 in assay buffer for 90 min at 4° C. Arrays were washed again and dried by centrifugation. Arrays were scanned at 10-μm resolution using a microarray scanner (Axon 4200AL with GenePix Pro Software; Molecular Devices), and fluorescence was detected according to the manufacturer’s instructions. Images were saved as 16-bit TIFF files and analysis was performed using GenePix. The median net intensity in relative fluorescence units (rfu) was reported for each spot. Out of 9,481 antigens, 77 cancer-testis antigens were selected and analyzed for pre- and post-vaccine expression in the sera (Table 4).

### Statistical analyses

All analyses were performed using GraphPad Prism ver.6.00 for Mac (GraphPad Software, CA, USA). The probability of survival was calculated by the Kaplan–Meier method, and statistical differences were evaluated by the log-rank test.

## SUPPLEMENTARY MATERIALS FIGURE AND TABLE



## Abbreviations

CHP: cholesteryl pullulan
MAGE: melanoma-associated antigen
MHC: major histocompatibility antigen complex
SD: stable disease
PD: progressive disease
OD: optical density
ELISA: enzyme-linked immunosorbent assay
cDNA: complementary DNA
PS: performance status
HIV: human immunodeficiency virus
RECIST: response evaluation criteria in solid tumors
CTCAE: common terminology criteria for adverse events
PCR: polymerase chain reaction
IgG: immunoglobulin G
BSA: bovine serum albumin
PBS: phosphate buffered saline
GST: glutathione S-transferase
SD: standard deviation
