# Supplementary material for: NY-ESO-1 antigen expression and immune response are associated with poor prognosis in MAGE-A4-vaccinated patients with esophageal or head/neck squamous cell carcinoma
Source: Oncotarget. 2018 Nov 13;9(89):35997–6011. doi: 10.18632/oncotarget.26323 (PMC6267599; doi:10.18632/oncotarget.26323)
Supplement: Supplementary file 1 [file oncotarget-09-35997-s001.pdf]

NY-ESO-1 antigen expression and immune response are associated with poor prognosis in MAGE-A4-vaccinated patients with esophageal or head/neck squamous cell carcinoma

SUPPLEMENTARY MATERIALS

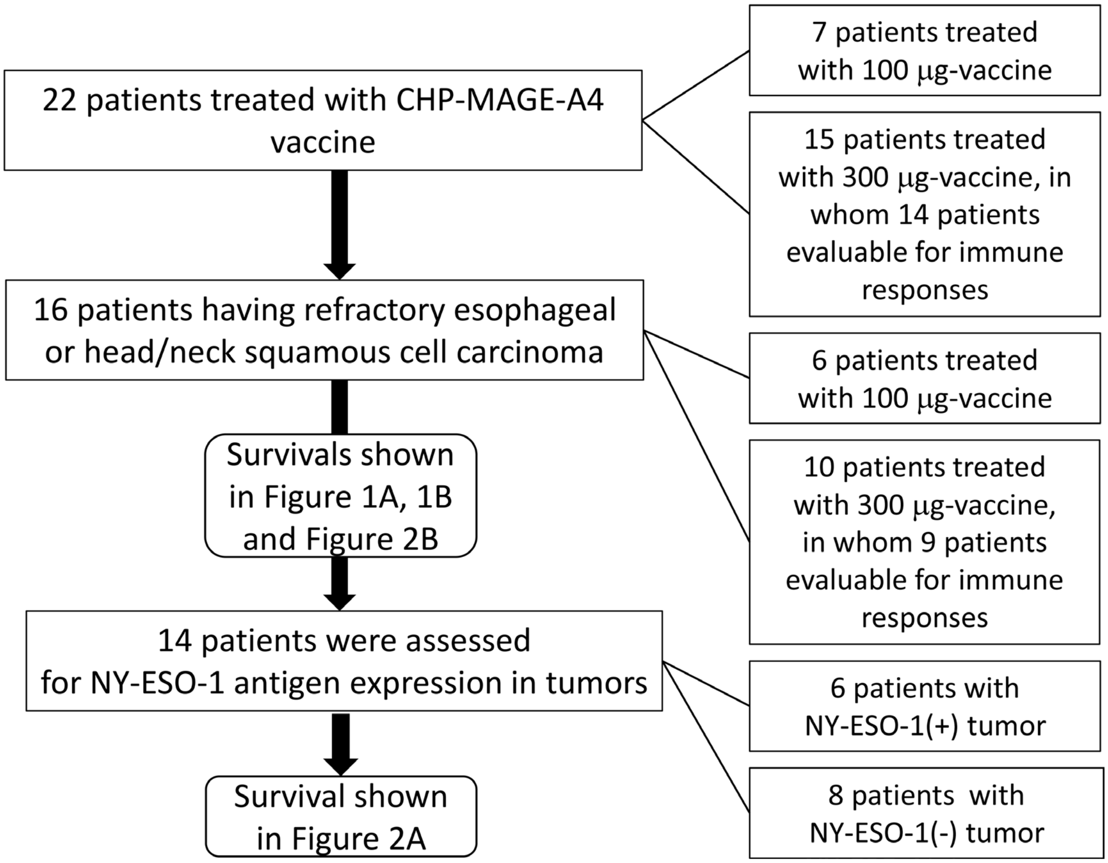

Supplementary Figure 1: Study profile. A total of 22 patients were enrolled to the clinical trial. Seven and 15 patients were treated with 100 μg-dese and 300 μg-dose of CHP-MAGE-A4, respectively, in whom 14 patients in the 300 μg-dose were evaluable for immune responses. 18 had esophageal or head/neck squamous cell carcinoma. Among them,16 patients had refractory tumors, and 2 had no tumors after the first-line therapy. We evaluated the 16 patients with refractory esophageal or head/neck squamous cell carcinoma for survival analysis. The 14 patients were assessed for NY-ESO-1 antigen expression in the tumor tissues.

Supplementary Table 1: Coexpression of NY-ESO-1 antigen in MAGE-A4 antigen-expressing tumors

pt No.	code No.	MAGE-A4 (PCR)	PCR copy number for MAGE-A4	NY-ESO-1 (PCR)	PCR copy number for NY-ESO-1	pt No	code No	MAGE-A4 (IHC)	IHC positivity for MAGE-A4(strength)	NY-ESO-1 (IHC)	IHC positivity (strength)
1	766	+	1171	–	0	12	KIT-5	+	MCV-1 90%(weak) MCV-4 95%(weak)	+	5%(weak)
2	630	+	320.85	+	1.84	13	KIT-8	+	MCV-1 30%(moderate) MCV-4 30%(weak)	+	30%(moderate)
3	887	+*	57B 60% MCV-1 40%	–*	nt	14	KIT-9	+**	15459.5**	–	negative
4	698	+	55.3	–	0	15	KIT-10	+	NA	–	negative
5	687	+	864	–	0	16	KIT-11	+	NA	NA	NA
6	998	+	900.3	–	0	17	KIT-12	+	NA	+	30%(moderate)
7	1147	+	4558.8	–	0	18	KIT-13	+	MCV-1 2%(weak) MCV-4 20%(weak)	+	70%(strong)
8	1358	+	375.1	–	0	19	KIT-14	+**	103.5	–	negative
9	1188	+*	5	–	0	20	KIT-15	+	MCV-1 30%(weak) MCV-4 negative	+	5%(weak)
10	704	+	2880.32	+	10.25	21	KIT-16	+	NA	–	negative
11	NMC001	+*	NA	NA	NA	22	KIT-17	+**	949.8	–	negative

*tested by IHC, **tested by PCR.
IHC, immunohistochemistry NA, not available.
